# Influence of *Limnospira platensis* Extract on Zootechnical and Immune Parameters of *Penaeus vannamei*

**DOI:** 10.3390/ani16172760

**Published:** 2026-09-02

**Authors:** Yosu Candela, Ignacio Jauralde, Luana Bortolini Giesta, Ivanilson Santos, David S. Peñaranda, Wilson Wasielesky, Marcelo Borges Tesser, Silvia Martínez-Llorens, Luís H. Poersch

**Affiliations:** 1 Aquaculture and Biodiversity Research Group, Institute of Animal Science and Technology, Universitat Politècnica de València, Camí de Vera s/n, 46022 Valencia, Spainivanilsonhp@gmail.com (I.S.);; 2 Aquatic Organism Immunology and Pathology Laboratory, Institute of Oceanography, Federal University of Rio Grande, Rio Grande 96210-030, RS, Brazil; 3 Laboratory of Aquaculture Impact Assessment, Institute of Oceanography, Federal University of Rio Grande, Rio Grande 96210-030, RS, Brazil; 4 Marine Shrimp Culture Laboratory, Institute of Oceanography, Federal University of Rio Grande, Rio Grande 96210-030, RS, Brazil; manow@mikrus.com.br; 5 Laboratory of Aquatic Organism Nutrition, Institute of Oceanography, Federal University of Rio Grande, Rio Grande 96210-030, RS, Brazil

**Keywords:** spirulina extract, *Arthrospira platensis*, immune response, *Litopeanaeus vannamei*, hemocytes, supplementation, bioactive, biocomponents, immunostimulants

## Abstract

This study evaluated the immunomodulatory and histological effects of dietary supplementation with an ethanol extract of *Limnospira platensis* (spirulina) in *Penaeus vannamei*. Shrimp were fed diets supplemented with 0.5%, 1%, 2%, and 4% spirulina extract for 35 days. Growth performance did not differ significantly among treatments, suggesting no adverse effects on nutrient utilization. However, immune parameters, including granulocyte abundance and hemocyte phagocytic capacity, improved significantly at 0.5–2% inclusion levels, with 0.5% promoting the highest granulocyte counts and 2% maximizing phagocytic activity. In contrast, the 4% treatment led to a decline in both granulocyte levels and phagocytosis, indicating saturation effects at higher doses. Histological analyses supported these findings, as villus length improved with 0.5, 1 and 2% doses but not with 4%. Overall, these results confirm a non-linear immunostimulatory response of *L. platensis* extract, highlighting low inclusion levels, especially 0.5%, as effective and economically feasible strategies to strengthen shrimp immunity in intensive aquaculture.

## Simple Summary

Shrimp farming is an important part of global aquaculture, but disease outbreaks and the stress of crowded farming conditions can seriously affect shrimp health and survival. Boosting shrimp’s natural immune defenses through diet is one way to help them resist disease without relying on antibiotics. Spirulina, a nutrient-rich microorganism already used in animal feeds, has shown promise for this purpose, but the right amount to include in shrimp diets is not well established. In this study, whiteleg shrimp were fed diets containing an alcohol-based spirulina extract at five levels (0%, 0.5%, 1%, 2%, and 4% of the diet) for 35 days, and their growth, immune cells, and gut tissue were examined. Shrimp grew normally on all diets, but those receiving low-to-moderate amounts of the extract, especially 0.5%, showed the strongest signs of improved immune activity and healthier intestinal tissue, while the highest dose (4%) did not provide the same benefits. These findings suggest that small amounts of spirulina extract are enough to support shrimp health, offering a practical, cost-effective way to strengthen disease resistance in shrimp farming.

## 1. Introduction

Aquaculture has expanded rapidly over recent decades, with shrimp farming representing one of the most economically valuable sectors of global aquaculture and playing a key role in international seafood trade [1]. However, this rapid expansion has also exposed the sector to major production challenges, particularly disease outbreaks such as white spot disease, which have caused severe economic losses worldwide [2]. As a result, shrimp farming is considered not only one of the most economically important sectors in aquaculture, but also one of the most vulnerable to losses associated with disease and environmental stress. Under intensive culture conditions, shrimp are frequently exposed to multiple environmental and physiological stressors, including high stocking densities, fluctuations in temperature and salinity, low dissolved oxygen levels, ammonia accumulation, handling stress, and pathogen pressure, that affect growth performance, impair immune function, and increase susceptibility to disease outbreaks, ultimately leading to significant economic losses in aquaculture production [3].

Therefore, increasing production must be accompanied by the sustainable development of methods and practices that minimize the impact of disease. Recommended practices to improve sustainability include improving the health of organisms and reducing the use of antibiotics [4].

Feed additives have proven useful for stimulating the primary immune system, improving intestinal health, stress tolerance, and disease resistance [5]. Unlike vertebrates, crustaceans lack immune memory, so stimulating the primary response is especially important in shrimp, in which the hemocytes play a central role [6]. The use of probiotics has proven very useful in controlling some diseases through what has been called immune system modulation [7]. However, many other compounds, either alone or in combination with probiotics, may stimulate the primary immune response, and plant and algae-based extracts have shown promising potential [4]. In this context, it is necessary to study the use of active biocomponents that help reduce antibiotic use and improve the immune response. Consequently, enhancing the innate immune response of shrimp has become a key strategy for improving health under intensive farming conditions [8].

Many studies have demonstrated that plant and herbal extracts can enhance innate immune function by promoting the total hemocyte count, phagocytosis, antibacterial activity, bacteriolytic activity, and resistance against pathogens [5,9]. Spirulina (*Limnospira platensis*, recently reassigned from *Arthrospira*) is a filamentous, photosynthetic cyanobacterium widely incorporated into aquafeeds; under the European Union regulatory framework, it is classified as a feed material rather than a feed additive [Commission Regulation (EU) No 68/2013]. Its dietary inclusion has been associated with enhanced total hemocyte counts and improved phagocytic and phenoloxidase activity in shrimp [10,11,12], alongside reported antioxidant and growth-promoting effects [13].

These effects have been attributed to several bioactive compounds present in spirulina biomass, including phenolic compounds such as flavonoids [14,15], the pigment-protein phycocyanin, associated with modulation of cytokine production and macrophage activity in *P. vannamei* [16], and sulfated polysaccharides such as spirulan, reported to have antiviral and immunomodulatory properties [17]. However, the relative contribution of each compound to the observed effects remains unclear, particularly when biomass is processed into an ethanol extract: phycocyanin, for instance, is primarily water-soluble, so its presence in a 96% ethanol extract cannot be assumed without specific biochemical characterization, and the concentration of polysaccharides may likewise differ substantially from that of whole biomass.

In shrimp, *L. platensis* supplementation significantly enhances the immunological status, particularly by improving the quality of hemolymph and modulating hemocyte activity. Hemolymph quality, as reflected by parameters such as total hemocyte count, hemocyanin concentration, and oxidative status, is crucial for maintaining shrimp health and resilience against pathogens [10]. Dietary inclusion of *L. platensis* has been associated with elevated total hemocyte counts and enhanced functional activities of hemocytes, including phagocytosis and phenoloxidase activity [11,12]. Improved hemocyte performance following *L. platensis* supplementation is thought to be associated with the microalga’s rich content of bioactive compounds, such as phycocyanin and polysaccharides, which modulate immune-related signaling pathways and protein expression in shrimp [9,18]. Nevertheless, the specific compounds responsible for these effects remain to be determined, particularly when ethanol-extracted preparations are used.

Immune-stimulating bioactives are, in addition, known to exert saturable, dose-dependent effects. Estimating the minimum effective dose required to elicit an immune response is therefore of critical importance, both because immunostimulant extracts represent a high cost relative to conventional dietary ingredients, and because their effects may vary considerably or even become detrimental, depending on the inclusion level. Moreover, high inclusion levels, such as those typically required when whole biomass rather than a concentrated extract is used, can significantly constrain diet formulation by displacing other nutritionally relevant ingredients, making them less desirable from a practical standpoint. Based on this rationale, we hypothesized that dietary supplementation with ethanol-extracted *L. platensis* would modulate the innate immune response and intestinal morphology of *P. vannamei* in a non-linear, dose-dependent manner, with immunostimulatory effects expected at low-to-moderate inclusion levels and an attenuation or reversal of these effects at higher doses, consistent with a saturation or regulatory feedback mechanism. To test this hypothesis, and to identify a dose range that is both immunologically effective and practically viable for diet formulation, the present study evaluated the effects of different inclusion levels (0.5%, 1%, 2%, and 4%) of ethanol-extracted *L. platensis* in commercial diets for *P. vannamei*, focusing on growth performance, innate immune response, and digestive tract histology.

## 2. Materials and Methods

### 2.1. Experimental Design

Shrimp with an initial weight of 2.05 ± 0.1 g were fed 5 diets: a control diet and 4 diets with different spirulina alcoholic-extract dietary inclusion (0.5, 1, 2 and 4% of spirulina extract, respectively) for 35 days.

The experiment was carried out at the facilities of the Marine Aquaculture Station (EMA) in an open clear-water system located in the maturation room of the Marine Shrimp Laboratory, Federal University of Rio Grande (FURG). Post-larvae *P. vannamei* were obtained from a commercial hatchery—Aquatec, Canguaretama, RN, Brazil.

15 Shrimp per tank were placed in 15 tanks (3 tanks for each treatment, 130 L per tank) for a period of 35 days, with sampling every 15 days to update biomass intakes correctly. Juvenile shrimp were maintained at a salinity of 30 psu and at a temperature of 27.7 ± 0.4 °C. The water exchange rate was 33% of the total tank volume per day.

### 2.2. Formulation and Manufacture of Experimental Diets

The ingredient compositions of experimental diets were made at the Laboratory of Aquatic Organism Nutrition (LANOA) according to the methodology of the Association of the Official Analytical Chemists [19].

Diets were formulated to contain approximately 39% crude protein and 9% fat with different levels of spirulina extract (0.5, 1, 2 and 4% of spirulina extract, respectively) and with a control (CON) diet without supplementation (Table 1).

To manufacture the diets, all ingredients were mixed in a feed mixer (Hobart Corporation, Troy, OH, USA) for 15 min, first the dry ingredients and afterwards with the liquid ingredients. After mixing, hot water was then blended into the mixture to attain a consistency appropriate for pelleting. Afterwards, spirulina extract was dissolved in distilled water and sprayed onto the mixture diets prior to pelleting. The same volume of distilled water was applied to the control diet to ensure consistent moisture levels. All inclusion dosages were calculated based on the dry matter content of the extract. Each diet was pressure-pelleted using a meat grinder (Metalúrgica 9000, PC-22, São Paulo, SP, Brazil) and a 2 mm die. After pelleting, the diets were dried to a moisture content of 8–10% using a forced air-drying oven (<40 °C) and stored at 4 °C. The pellets were broken down to the desired diameter (1 mm).

### 2.3. Spirulina Extract

The method proposed by Amaral [20] with some modifications was used to prepare *L. platensis* extract. This method was performed in the facilities of the Laboratory of Biochemical Engineering, College of Chemistry and Food Engineering at FURG. In this method, 1500 mL of 96% ethanol was added to 300 g of powdered *Limnospira platensis*, and this solution was kept in the refrigerator for 72 h. Then, 250 mL of distilled water was added to the above solution and blended for 15 min. Following filtering the solution by Whatman paper, the obtained liquid was placed in a water bath to remove the alcohol. The extracted solution was poured into two 1000 mL Erlenmeyer flasks and placed in an incubator at 41 °C until its volume reached 500 mL in each Erlenmeyer flask (during 3 days). Finally, the effective extract (0.43 g/mL) was obtained by drying the extract. Then, 23.25 mL (equivalent to the extract) was added to 2 kg feed as group SP05, 46.50 mL to 2 kg feed as group SP1, 93 mL to 2 kg as group SP2 and 186 mL to 2 kg as group SP4. The extract was not chemically characterized in the present study (see Section 4 for a discussion of this limitation and recommendations for future work).

### 2.4. Growth Trial

Juvenile shrimp began the trial with a mean weight of 2.05 g ± 0.1. All shrimp were sampled every 15 days to update biomass and estimate feed intakes correctly.

The growth was estimated using the Specific Growth Rate parameter (SGR) following the equation:

SGR = 100 × (LN(BWf) − LN(BWi))/days

where the initial body weight is BWi, BWf is the final body weight, and the number of days between both weights.

For feed conversion ratio (FCR) and protein efficiency ratio (PER), the final biomass was corrected to consider the biomass of dead shrimp during the period:

Final biomass = Final number of shrimp × Final weight + Estimated biomass of dead shrimp.

The biomass of dead shrimp was estimated with the average weight between sampling periods and the number of dead shrimp.

Protein efficiency ratio = (wet weight gain/protein intake).

Survival = (final number of shrimp/initial number of shrimp) × 100.

The health status of the shrimp was monitored daily, and they were fed at a restricted feeding rate of 2.7 g/100 g of shrimp/day. Diet was proportioned twice per day, at 8 a.m. and at 5 p.m. The feeding was divided into two daily intakes (morning and afternoon) and was performed manually using feeding trays.

### 2.5. Measurements of Immune Parameters

#### 2.5.1. Hemocyte Differentiation

At the end of the trial, 5 shrimp were collected from each experimental unit. The hemolymph (approximately 45 μL per tank) was withdrawn from the heart using a Hamilton syringe (50 μL) and was transferred directly into an anticoagulant solution (1:4) (modified Alsever’s solution or MAS: 27 mM sodium citrate, 336 mM sodium chloride, 115 mM glucose, 9 mM EDTA, pH 7.0). After this, a drop of sample was placed on top of a slide to spread the hemolymph and fixed in methanol for 10 min. Regarding the staining of the samples, two stains were used: Giemsa and May–Grünwald. Once the slides were dried, they were placed in darkness for 2 h.

For the hemocyte counting and differentiation protocol, the slides were divided into 10 areas, with about 25 hemocytes per area. Two types of hemocytes were differentiated: granulocytes and hyalinocytes. The hemocyte count was performed on a microscope eyepiece, including 5 lines and 25 points (Carl Zeiss do Brasil, São Paulo, Brazil) according to [21]. Another 10 μL hemolymph was used to determine the total protein concentration (TPC) in the shrimp serum; TPC was determined according to [22].

#### 2.5.2. Phagocytic Capacity

To analyze the phagocytic capacity (PC), 3 samples from each tank were used, totaling 45 samples. 10 μL of hemolymph was removed from each chamber and mixed with 10 μL of mild solution for PC (7.5% mild, 0.89% red and PBS). The mixture was placed on a slide to carry out the smear and then fixed in methanol for 10 min. This method has been adapted and developed from [23,24] protocol.

The slides were divided into 4 fields for counting using a light microscope. Phagocytic hemocytes and the total number of hemocytes were counted. From this point, the calculation was performed, presenting the data as a percentage.

Phagocytic capacity (PC %) = (total number of phagocytic hemocytes × 100) × (number of total hemocytes) − 1

#### 2.5.3. Histology Analysis

At the end of the experimental period, shrimp were collected and immediately transferred to containers with chilled seawater. Following histology protocol [25], the shrimp were fixed in Davidson’s solution (11.5% acetic acid, 22% formalin, and 33% ethanol at 96%) by perfusion into the cephalothorax and abdominal segments and immersion for 24 h. After fixation, samples were transferred to 70% ethanol and stored until histological processing.

For histological analysis, tissue samples were segmented and processed using an automatic tissue processor (Leica TP1020, Leica Biosystems, Nussloch, Germany) and embedded in Paraplast^®^ (Leica Biosystems, Nussloch, Germany). Histological sections (5 μm thickness) were prepared using a semi-automatic rotary microtome (Leica RM2245, Leica Biosystems, Nussloch, Germany), mounted on frosted-end glass slides, deparaffinized, and stained with hematoxylin and eosin for examination under a light microscope. Descriptive histopathological observations were performed for each organ, and quantitative analyses were conducted following the procedures described by Li et al. [26].

In the hepatopancreas, the tissue-to-lumen area ratio was measured, and counts of R and B cells were performed according to the methodologies of Cervelloni et al. and Jahan et al. [25,27]. Additionally, intestinal villi height was quantified to assess potential morphological changes associated with the dietary treatments. The villi were measured from their base, where they join the mucosa, to their tip, which projects into the lumen of the intestine.

The quantitative histological analysis provided insights into the structural condition of hepatopancreatic and intestinal tissues, allowing the evaluation of possible alterations related to *Limnospira platensis* extract supplementation. These histological parameters were selected as sensitive indicators of tissue integrity and metabolic activity in *P. vannamei*, as previously validated in comparable studies [25,27].

### 2.6. Statistical Analysis

All data were expressed as mean ± standard deviation (SD). Prior to statistical analysis, the assumptions of normality and homogeneity of variance were evaluated using the Shapiro–Wilk and Levene tests, respectively. Differences among dietary treatments were analyzed by one-way analysis of variance (ANOVA), followed by Tukey’s honestly significant difference (HSD) test for multiple comparisons when significant differences were detected. Percentage and proportion data, including granulocyte and hyalinocyte proportions, phagocytic capacity, and survival rate, were subjected to arcsine square-root transformation when required to meet ANOVA assumptions. Statistical significance was considered at *p* < 0.05. Statistical analyses of the data were performed with Statgraphics, Statistical Graphics System, version Centurion 18-X64.

## 3. Results

The growth performance and survival of *P. vannamei* fed the experimental diets for 35 days are summarized in Table 2. No significant differences were found in the final weight. Final weights ranged from 6.41 g with the SP4 treatment to 7.23 g for the SP05 treatment. Specific growth rate, feed conversion ratio, and protein efficiency ratio also did not show significant differences among treatments. SGR means per treatment ranged from 3.00% day^−1^ for shrimp fed the SP4 diet to 3.38% day^−1^ for the shrimp fed the SP1 diet, while FCR varied between 1.54 and 1.97 for the shrimp fed the SP05 and SP4, respectively. Survival remained high across all treatments (84.44 to 93.33%). The Weekly Gain data also showed no significant differences among treatments. Shrimp fed the SP05 and SP1 (0.99 g week^−1^) diets exhibited the highest weekly weight gain, whereas the SP4 group showed the lowest value (0.83 g week^−1^).

The distribution of hemocyte types in shrimp fed with different diets is presented in Table 3. Shrimp fed with diets with different inclusions of spirulina extract showed significant differences in the percentage of granulocytes and hyalinocytes in all treatments. The hemolymph of the shrimp fed with a diet containing a 0.5% spirulina extract, SP05, exhibited the highest proportion of granulocytes, 91.44% and consequently the lowest percentage of hyalinocytes, 8.56%, while the lowest percentage of granulocytes was shown by the shrimp fed with the control diet, 23.05% and the corresponding 76.95% of hyalinocytes.

**Figure 1:**
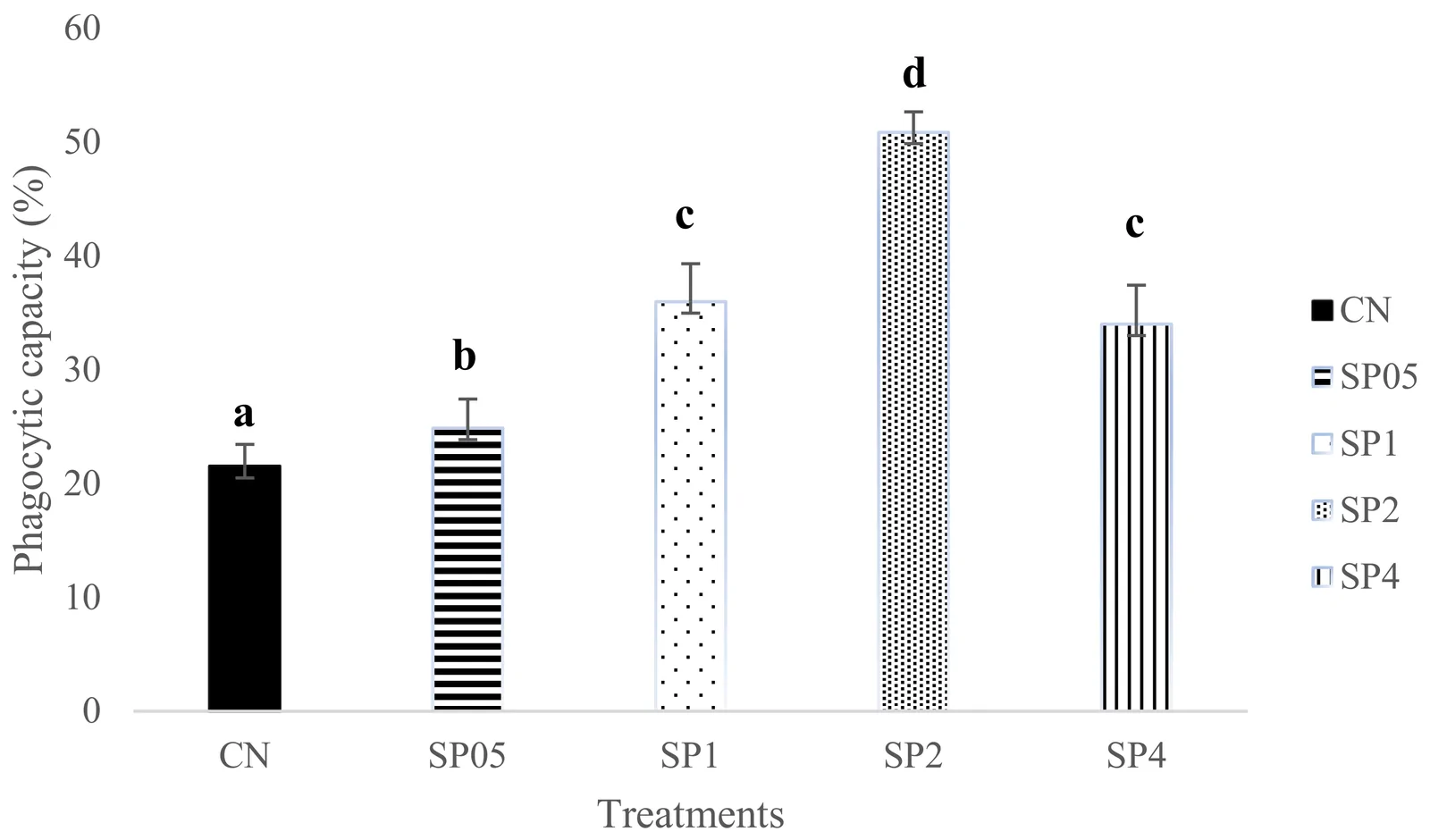
Phagocytic capacity (PC %) of hemocytes in shrimp hemolymph from all experimental treatments. Values shown in the graphic are the mean (*n* = 3) ± standard deviation. *p*-value <0.0001. Different letters in the same row indicate significant statistical differences (*p* < 0.05). Newman–Keuls test.

The phagocytic capacities of hemocytes are shown in Figure 1. All treatments containing *L. platensis* extract exhibited significantly higher phagocytic capacity values than the control group, with a value of 21.49. The highest phagocytic capacity was recorded for the shrimp fed with the SP2 diet (50.84%), followed by those fed with SP1 (35.96%), SP4 (34.00%), and SP05 (24.85 ± 2.56%).

Regarding histological analysis, significant differences were observed in the morphology of intestinal samples among treatments (Table 4). Shrimp fed with SP05, SP1, and SP2 diets exhibited significantly higher villus height than either the control or SP4 groups (*p* < 0.05). Furthermore, villus height in the SP4 group was significantly lower than in the control group (*p* < 0.05).

R- and B-cell counts of hepatopancreas samples can be seen in Table 5. In the hepatopancreas, experimental groups with spirulina extract presented significantly lower proportions of B-cells than the control group (*p* < 0.05). SP2 and SP1 showed the lowest B-cell abundance compared with the control, which showed the highest B-cell value. Very similar differences were observed in R-cell results, in which inclusion diets (SP05, SP1, SP2 and SP4) showed statistically lower values than the control diet.

**Figure 2:**
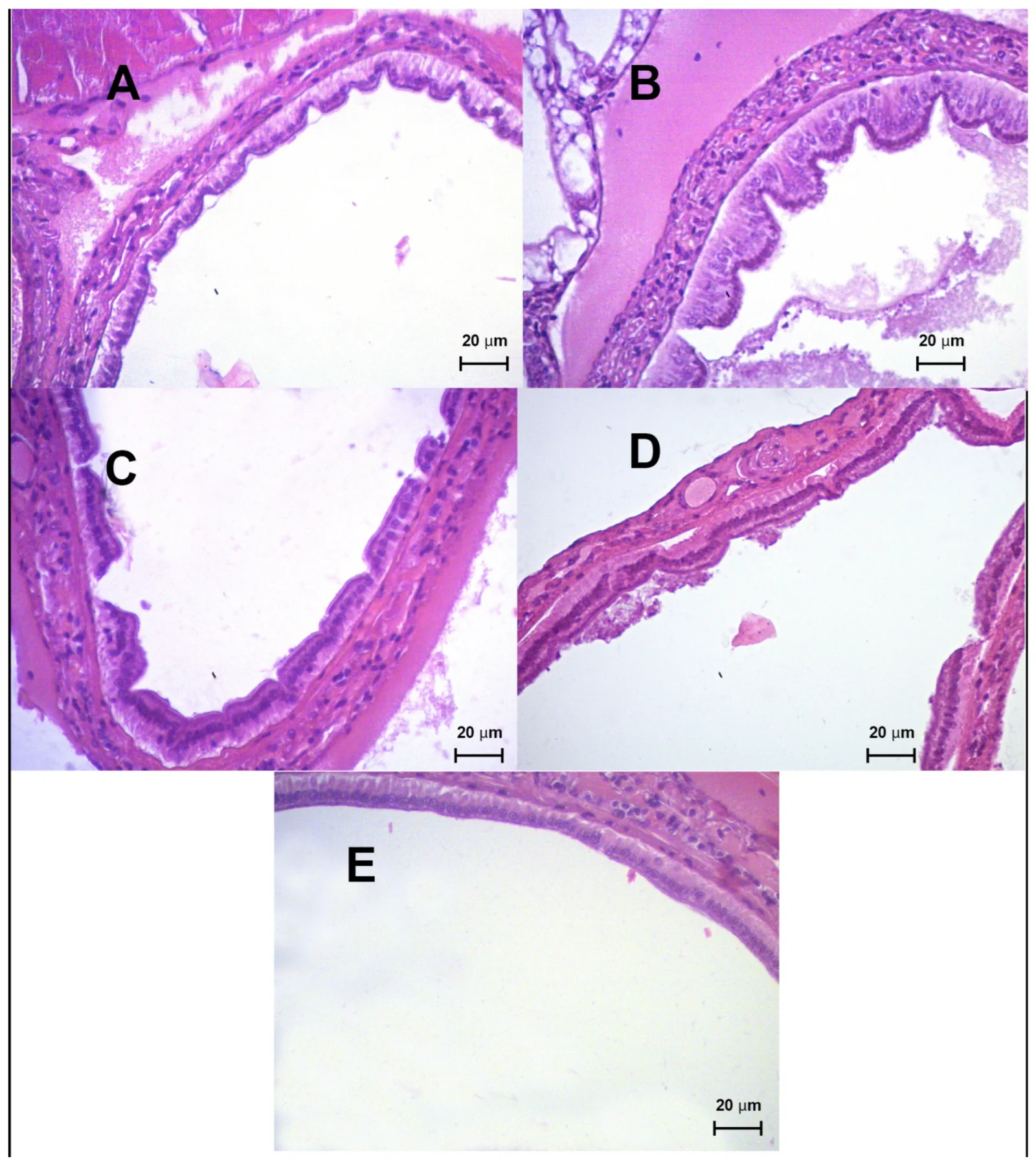
Microscopic histological images (40×) of *P. vannamei* intestine, stained with hematoxylin and eosin, fed with experimental diets. CN = (**A**), SP05 = (**B**), SP1 = (**C**), SP2 = (**D**) and SP4 = (**E**).

**Figure 3:**
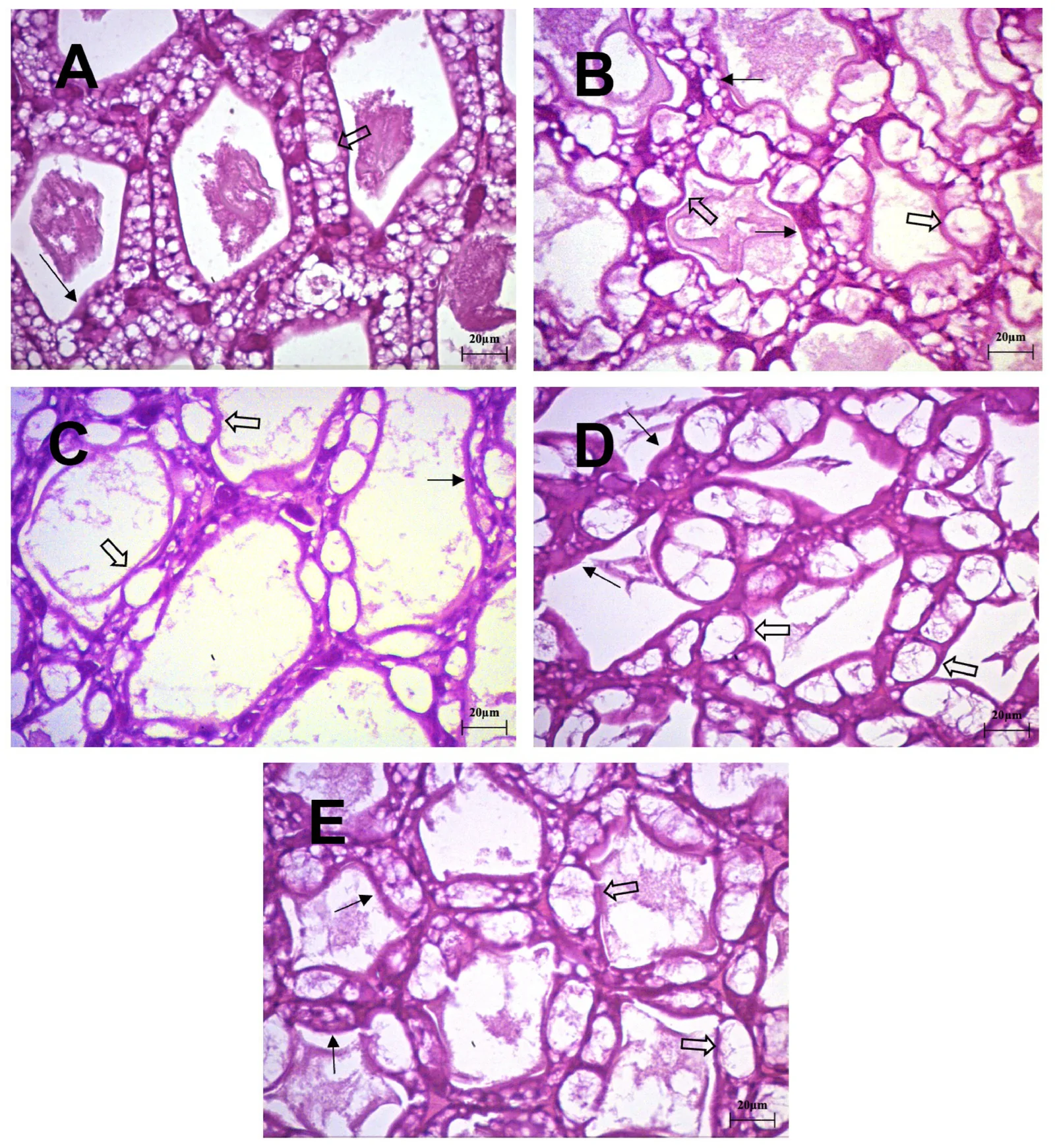
Microscopic histological images (40×) of *P. vannamei* hepatopancreas, stained with hematoxylin and eosin, fed with experimental diets. CN = (**A**), SP05 = (**B**), SP1 = (**C**), SP2 = (**D**) and SP4 = (**E**). Thick Arrows = B-cells and thin arrows = R-cells.

Quantitative analysis of both intestine and hepatopancreas samples can be seen in Figure 2 and Figure 3, respectively.

## 4. Discussion

*Limnospira platensis* has been widely investigated as a potent immunostimulant in shrimp through diverse experimental approaches, including dietary supplementation, bioactive fractions, and extract-based applications. Numerous studies have demonstrated that spirulina can enhance growth, immune activity, and resistance to pathogens in *Penaeus vannamei*, whether provided as a meal substitute, hot-water extract, alcoholic extract, or purified phycocyanin. These effects have been consistently reported across meta-analytic evidence and targeted experimental trials using oral, immersion, or injection routes [10,13,16,28,29,30]. Collectively, these studies highlight the versatility of spirulina as a functional feed additive for improving shrimp health. However, most existing studies have not identified a threshold beyond which the immunostimulatory benefits may decline. In this context, our findings suggest the existence of an optimal inclusion level for the immunostimulatory effects of *L. platensis* extract, beyond which immune responses declined, as reflected by the decline in granulocyte proportions, phagocytic activity, and villus height at the highest tested dose (4%).

In the present study, the inclusion of *L. platensis* extract in shrimp diets did not significantly affect growth performance among treatments (*p* > 0.05). Survival rates remained high across treatments, ranging from 84.4% to 93.3%, while feed conversion ratios (FCR) varied narrowly between 1.54 and 1.97, showing no significant variation among groups. The feeding trial lasted only 35 days, which constitutes a limitation for fully assessing long-term zootechnical performance in sub-adult *Penaeus vannamei*. Although no significant differences in growth parameters were detected among treatments under the experimental conditions evaluated, a longer feeding trial, such as 8 weeks or more, would be required to better determine the sustained effects of *L. platensis* extract on growth performance, feed utilization, and physiological responses.

Previous studies have reported variable effects of spirulina supplementation on shrimp growth performance depending on its form of application and dietary context. Spirulina powder has been successfully used as a functional ingredient to partially replace fishmeal, with inclusion levels of up to 75% reported without negative effects on growth [31]. Other studies attribute an enhancement of growth performance when spirulina was included at lower levels as a dietary additive or as a partial substitute for soybean meal [32]. However, no clear enhancement of growth has been reported in a meta-analysis when it is used as a functional ingredient [13]. These contrasting results suggest that the beneficial effects of spirulina on growth may depend more on its role as a functional additive than as a major dietary ingredient. It seems more likely that the effect occurs when used as an additive (as spirulina powder) [33] than with the inclusion of spirulina meal as a full ingredient, which the literature recommends between 15 and 25% of spirulina. [13,28,32]. When fishmeal is extensively replaced by spirulina meal, the bioactive compounds present in the microalga, such as the antioxidants, phycocyanins and polysaccharides, may not compensate for the negative effects of fishmeal substitution. This would theoretically make extracts a better candidate for improving growth parameters, as improved shrimp growth has been reported in other studies when extracts are used [13,16,34]. Other reports suggest that while *L. platensis* can act as a functional ingredient, its zootechnical effects may be limited when basal diets are already nutritionally balanced and husbandry conditions are optimal, giving better results when it is used as an additive or the extracts of phycocyanin [16].

In the present study, the addition of spirulina extract did not improve growth results. A possible explanation is that the experimental diets were already nutritionally balanced and formulated with relatively low levels of potentially pro-inflammatory plant ingredients. Under such conditions, the anti-inflammatory and gut-regulatory effects attributed to spirulina bioactive compounds may have limited impact on nutrient utilization and growth. A closer examination of the results suggests that when experimental diets contain low levels of pro-inflammatory components, such as plant-based meals, the replacement with spirulina does not enhance growth but rather maintains it [31]. In contrast, improvements in growth performance have been reported when spirulina replaces soybean meal specifically [32]. Similarly, Novriadi et al. [35] observed enhanced growth in shrimp fed diets with potential pro-inflammatory ingredients. An exception is noted in Ahmed et al. [28], who reported improved growth performance using very low inclusion levels (0.4%) of spirulina powder in diets formulated with minimal plant-derived components.

Despite the absence of a growth response, immune modulation was evident, particularly in the proportion of granulocytes and the phagocytic capacity of hemocytes. Shrimp fed diets containing *L. platensis* extract showed a strong shift in hemocyte profile, characterized by an increased percentage of granulocytes and decreased hyalinocytes. Granulocytes are the primary effector cells in crustacean innate immunity: they contain abundant cytoplasmic granules packed with antimicrobial peptides such as penaeidins, which are produced and stored almost exclusively in this hemocyte lineage and released upon microbial challenge [36]. These antimicrobial peptides (penaeidins) exhibit strong antibacterial and antifungal activities and participate directly in pathogen elimination, making granulocytes crucial for the first line of defense. In addition, granulocytes contribute to phagocytosis, encapsulation, and prophenoloxidase activation pathways. Although increased granulocyte abundance and phagocytic activity are commonly associated with infection, in the absence of pathogenic challenge they are generally interpreted as indicators of enhanced immune competence [37]. Therefore, an increase in granulocyte proportion, as observed in our SP05 and SP1 and SP2 treatments, indicates enhanced immunological readiness.

Comparable increases in granulocyte abundance have previously been reported in shrimp treated with spirulina hot-water extracts administered by immersion [10,29], as well as with dietary supplementation of concentrated phycocyanin derived from *L. platensis* [16]. These immunostimulatory effects are associated with bioactive compounds present in spirulina, including phycocyanin, β-carotene, and sulfated polysaccharides, which have been shown to activate the prophenoloxidase cascade, stimulate reactive oxygen species production, and regulate immune-related pathways in crustaceans [8,12]. However, the present study employed a 96% ethanol extract that was not quantitatively characterized; therefore, the observed immune responses cannot be attributed specifically to individual compounds such as phycocyanin or polysaccharides. The biological effects observed are more cautiously interpreted as potentially associated with the complex mixture of ethanol-soluble bioactive compounds present in the extract.

Flavonoids have demonstrated immunoprotective effects in shrimp, notably enhancing resistance against *Vibrio* infections. In *Penaeus vannamei*, dietary supplementation with flavonoid-rich *Agave lechuguilla* extract [38] improved survival rates following pathogenic challenge. The bioactive compounds acted as modulators of immune responses and reduced bacterial load. These findings support the potential of flavonoid-based additives as natural disease-control strategies in aquaculture. Although derived from a terrestrial plant, similar flavonoid mechanisms may underlie the antimicrobial properties of spirulina-based supplements. Several studies indicate that extract-based applications of spirulina may provide stronger or more targeted immunostimulatory effects than whole-powder supplementation, likely because solvent extraction concentrates specific bioactive molecules [30]. However, the dose–response measurements found so far could indicate that higher doses, such as those in the present study, could optimize the response. Although [34] finds a quadratic relationship between the immune response in tilapia and the concentration of phycocyanin, it does not reach a maximum.

A limitation of the present study is the lack of a detailed biochemical characterization of the ethanol extract used. Future studies should include quantitative analyses of total phenolics, total flavonoids, and chromatographic profiling (e.g., HPLC or GC-MS) to identify the specific bioactive compounds responsible for the immunomodulatory effects observed in *Penaeus vannamei*.

Regarding phagocytic capacity, all supplemented treatments (SP05, SP1, SP2, and SP4) showed significantly higher values than the control diet. Although SP05 exhibited the highest granulocyte proportion, maximum phagocytic activity was observed in the SP1 and SP2 groups. These findings indicate that dietary supplementation with *L. platensis* extract enhanced both granulocyte abundance and phagocytic function, although the optimal response differed between immune parameters, suggesting distinct dose–response thresholds within the innate immune system. A comparable enhancement in phagocytic capacity has been reported by Chen et al. [8], who found that *Penaeus vannamei* fed diets enriched with *Limnospira platensis* dried powder displayed significantly higher phagocytic activity when challenged with *Vibrio alginolyticus*. Similarly, Lee et al. [39] demonstrated that *Penaeus merguiensis* receiving dried *L. platensis* at 0.3% dietary inclusion showed a marked increase in hemocyte phagocytosis. More recently, Sherif et al. [40] observed comparable immune stimulation in Nile tilapia fed fermented spirulina, reflected by higher phagocytic activity and antioxidant status. Collectively, these findings indicate that dietary supplementation with *L. platensis* extract modulates hemocyte-mediated immune parameters in a nonlinear dose-dependent manner, with the strongest responses observed at moderate inclusion levels. Interestingly, the immunostimulatory response declined at higher inclusion levels (4%), suggesting that excessive supplementation may trigger regulatory or inhibitory effects on immune activity. The immunosuppressive effect observed at the highest inclusion level (4%) may be explained by a phenomenon analogous to high-dose immune tolerance, whereby excessive or prolonged exposure to an immunostimulant leads to a hyporesponsive state rather than continued activation. This is consistent with the mechanism described by Smith et al. [41] in crustaceans, whereby non-self challenge triggers degranulation and lysis of granular and semigranular haemocytes, resulting in a transient reduction in circulating haemocyte numbers; the same authors reported that prolonged dietary glucan supplementation reduced survival in *Litopenaeus vannamei* relative to unsupplemented controls, illustrating that excessive or sustained immune stimulation can become detrimental rather than protective. A comparable dose-dependent switch from immune priming to tolerance, mediated by receptor desensitization rather than loss of recognition, has also been described in vertebrate innate immune cells exposed to high doses of pathogen-associated molecular patterns. Taken together, these findings suggest that at 4% inclusion, the immune system of *P. vannamei* may enter a refractory or exhausted state, consistent with the decline in granulocyte proportions and phagocytic activity observed in our study. Several authors have reported similar non-linear responses in aquatic species. For instance, Abdel-Tawwab and Ahmad [42] reported an asymptotic dose–response curve of phagocytic activity, measured indirectly, with the increase in dose of spirulina meal until 10% of inclusion.

The intestinal villi height results (Table 4) are consistent with a possible anti-inflammatory response to spirulina extract at inclusion levels between 0.5 and 2%, consistent with previous reports linking microalgal supplements to intestinal barrier protection and immune modulation [43]. Intestinal histology results further support the beneficial effects of dietary spirulina extract. Shrimp fed with SP05, SP1, and SP2 diets exhibited significantly greater villus height than the control and SP4 treatments, suggesting improved digestive function and better intestinal integrity. Similar enhancements in villus height and intestinal morphology have been observed in shrimp fed low levels (0.5–1%) of *L. platensis* meal. Prabu et al. [44] documented no abnormalities in intestinal histology when the diet was supplemented with spirulina powder at 1%. Likewise, dietary astaxanthin has been shown to promote epithelial cell proliferation and improve villus organization in the intestinal mucosa of crustaceans [45], supporting our findings of improved intestinal health with supplemented diets. Altogether, these histological outcomes suggest that inclusion levels of 0.5% to 1% of *L. platensis* extract are associated with favorable morphological changes in digestive organs, which may support digestive function; whether this translates into improved resilience against pathogen challenge remains to be tested. It should be noted, however, that although villus height was significantly altered by dietary treatment, the functional relevance of the intestine in shrimp is comparatively limited, since the majority of digestive and absorptive processes in decapod crustaceans take place in the hepatopancreas rather than in the intestine [46,47]. Therefore, while changes in villus morphology may reflect local mucosal responses to dietary spirulina extract, their physiological impact on overall nutrient digestion and absorption should be interpreted with caution. However, future studies should incorporate validated histopathological scoring methods to provide a more comprehensive assessment of intestinal and hepatopancreatic tissue alterations.

The reduced immune response observed in our experiment at the highest inclusion level (SP4) suggests the existence of an optimal inclusion threshold for the immunostimulatory effects of *L. platensis* extract. One possible explanation is that the high concentrations of spirulina antioxidant pigments, particularly phycocyanins, may saturate immune receptors or interfere with redox-sensitive signaling, thereby limiting hemocyte activation rather than enhancing it [35]. This mechanism helps explain the reduced phagocytic response observed in the SP4 treatment, consistent with previous findings showing compensatory downregulation of immune pathways when bioactive inputs exceed optimal thresholds [12,48]. Thus, while *L. platensis* extract acts as an effective immunostimulant at low to moderate doses, exceeding this range may attenuate immune activation, underscoring the importance of optimizing inclusion levels. This pattern is also evident in tilapia fed with phycocyanin extract [34], where a quadratic effect was reported for both growth and immune parameters in shrimp fed diets supplemented with phycocyanin extract. Specifically, in this study, tilapia growth performance plateaued at an inclusion level of 1.5%, whereas immune parameters continued to improve, reaching their peak at the highest tested concentration. In our study, the absence of significant improvements in shrimp growth, despite favorable immunological outcomes, may reflect a lack of intestinal inflammation. Notably, Maximum stimulation of granulocyte production was observed at a 0.5% inclusion level, while phagocytic activity peaked at 2%. However, the lowest immune response detected at the highest inclusion level (4%) suggests that excessive doses may reduce the immunostimulatory effect of the extract.

From a practical perspective, results of low inclusions (SP05 and SP1) were sufficient to enhance innate immunity and offer important economic and environmental advantages. The economic feasibility of *L. platensis* extract supplementation should be interpreted cautiously. Assuming a wholesale price of €10 kg^−1^ for *L. platensis*, the additional supplementation cost was estimated at approximately 0.05 € kg^−1^ feed for the 0.5% diet, 0.10 € kg^−1^ feed for the 1% diet, 0.20 € kg^−1^ feed for the 2% diet, and 0.40 € kg^−1^ feed for the 4% diet.

On a commercial scale, functional additives must balance cost and efficacy. Since *L. platensis* extract remains relatively costly compared with conventional sources, demonstrating strong immune benefits at minimal inclusion rates is particularly valuable [49,50]. As noted by Ragaza et al. [49], the large-scale application of spirulina is primarily limited by production costs related to cultivation and processing. Achieving measurable health and immune improvements at low inclusion levels improves economic feasibility, preserves feed palatability, and simplifies integration into commercial feed systems. Keeping additive levels below 1% significantly reduces expenses while achieving the immunomodulatory benefits described above, a desirable trait in intensive shrimp farming; whether these benefits translate into prophylactic protection against pathogens remains to be confirmed through dedicated challenge trials [9,48,50]. Moreover, these low-dose strategies align with current trends in functional aquafeeds emphasizing immunonutrition, eco-efficiency, and cost-effectiveness [49]. In conclusion, the present study demonstrates that ethanol-extracted *Limnospira platensis* acts primarily as an immunomodulatory additive in *Penaeus vannamei*.

## 5. Conclusions

Our findings show that 0.5%, 1%, and 2% extract inclusions consistently improved immune function, while no significant differences in growth performance were detected among treatments during the 35-day experimental period. Among the tested treatments, the 0.5% inclusion produced the strongest increase in granulocyte proportion, whereas the 2% inclusion yielded the highest phagocytic activity, indicating that different immune parameters may exhibit distinct optimal response thresholds. The decline in granulocyte proportions, phagocytic capacity, and villus height at the highest dose (4%) further supports the existence of a dose-dependent saturation threshold in the immunostimulatory response to *L. platensis* extract. Therefore, low inclusion rates of *L. platensis* extract represent a promising and economically viable strategy to enhance shrimp resilience in intensive culture systems; however, longer feeding trials are required to confirm their long-term effects on zootechnical performance.

## Figures and Tables

**Table 1:** Formulation and proximate composition of the experimental diets.

Ingredients (%)	Control	SP05	SP1	SP2	SP4
Fishmeal ^a^	35	35	35	35	35
Soybean meal ^b^	26	26	26	26	26
Corn starch ^c^	22	22	22	22	22
Wheat bran ^d^	10	10	10	10	10
Fish oil ^e^	2.4	2.4	2.4	2.4	2.4
Cellulose ^f^	3.6	3.6	3.6	3.6	3.6
Vitamin mineral-premix ^g^	1.0	1.0	1.0	1.0	1.0
Spirulina Supplementation					
*L. platensis* extract	0	0.5	1.0	2.0	4.0
Composition dry matter (%)					
Crude protein	38.84 ± 0.17	38.98 ± 0.17	39.16 ± 0.17	39.46 ± 0.17	40.05 ± 0.17
Crude lipid	9.21 ± 0.14	9.17 ± 0.14	9.14 ± 0.14	9.07 ± 0.14	8.94 ± 0.15
Crude fiber	5.47 ± 0.10	5.44 ± 0.11	5.42 ± 0.10	5.38 ± 0.10	5.29 ± 0.12
Dry matter	92.90 ± 0.12	92.91 ± 0.12	92.93 ± 0.12	92.96 ± 0.12	93.00 ± 0.10
Ash	11.65 ± 0.30	11.64 ± 0.30	11.65 ± 0.30	11.63 ± 0.29	11.63 ± 0.28

^a^ PETFAR, Nova Bréscia, RS, Brazil; ^b^ Sulino, São José do Hortêncio, RS, Brazil; ^c^ Tok, Vogel Alimentos, Canoas, RS, Brazil; ^d^ MOTASA, Taquari, RS, Brazil; ^e^ Campestre, São Paulo, SP, Brazil; ^f^ Synth ^®^, Labsynth, Diadema, SP, Brazil; ^g^ Tectron, Toledo, PR, Brazil; Vitamin A (min.) 2000 UI/kg, vitamin D3 (min.) 640,000 UI/kg, vitamin E (min.) 2400 UI/kg, vitamin K3 (min.) 680 mg/kg, vitamin B1 (min.) 400 mg/kg, vitamin B2 (min.) 1000 mg/kg, vitamin B6 (min.) 1200 mg/kg, vitamin B12 (min.) 4000 mcg/kg, niacin (min.) 9000 mg/kg, pantothenic acid (min.) 3000 mg/kg, folic acid (min.) 400 mg/kg, biotin (min.) 35 mg/kg, manganese (min.) 14 g/kg, zinc (min.) 11 g/kg, iron (min.) 10 g/kg, copper (min.) 2000 mg/kg, iodine (min.) 200 mg/kg, cobalt (min.) 40 mg/kg, selenium (min.) 40 mg/kg.

**Table 2:** Growth parameters (Final weight, survival, SGR, weekly gain, FCR and PER) of the 35-day experimental trial.

Parameters	Control	SP05	SP1	SP2	SP4	*p*-Value
Initial weight (g)	2.16 ± 0.15	2.27 ± 0.09	2.17 ± 0.17	2.10 ± 0.10	2.23 ± 0.09	0.5712
Final weight (g)	6.89 ± 0.50	7.23 ± 0.70	7.11 ± 0.69	6.44 ± 0.96	6.41 ± 0.81	0.5767
Survival (%)	88.9 ± 3.85	93.3 ± 6.67	84.4 ± 15.40	88.9 ± 3.85	93.3 ± 0.00	0.6433
SGR (%/day)	3.31 ± 0.03	3.30 ± 0.24	3.38 ± 0.14	3.18 ± 0.44	3.00 ± 0.38	0.5369
Weekly Gain (g/week)	0.94 ± 0.07	0.99 ± 0.13	0.99 ± 0.11	0.87 ± 0.19	0.84 ± 0.16	0.5767
FCR	1.77 ± 0.18	1.54 ± 0.06	1.64 ± 0.14	1.96 ± 0.18	1.97 ± 0.40	0.1503
PER	1.72 ± 0.18	1.97 ± 0.08	1.85 ± 0.15	1.56 ± 0.15	1.58 ± 0.29	0.0860

Values represented as mean ± standard deviation (*n* = 3). Different letters in the same row indicate significant statistical differences (*p* < 0.05). The means comparison test used was Student–Newman–Keuls. Specific Growth Rate (SGR) = 100 × ln (final weight/initial weight)/days. Weekly Gain (WG) = (final weight − initial weight)/weeks. Feed conversion ratio (FCR) = feed consumption (g)/biomass gain (g). PER = biomass gain (g)/protein intake (g).

**Table 3:** Differentiation of hemocytes from the hemolymph of shrimp fed the experimental diets.

Hemocytes (%)	CN	SP05	SP1	SP2	SP4	*p*-Value
Granulocytes	23.05 ^e^ ± 1.23	91.44 ^a^ ± 0.76	83.58 ^b^ ± 0.89	80.50 ^c^ ± 1.39	52.79 ^d^ ± 1.39	<0.0001
Hyalinocytes	76.95 ^e^ ± 1.23	8.56 ^a^ ± 0.76	16.42 ^b^ ± 0.89	19.50 ^c^ ± 1.39	47.21 ^d^ ± 1.13	<0.0001

Values are the mean (*n* = 3) ± standard deviation. Different letters in the same row indicate significant statistical differences (*p* < 0.05). Newman–Keuls test.

**Table 4:** Villus height (μm) from intestinal histological samples of shrimp fed with experimental diets.

Treatment	CN	SP05	SP1	SP2	SP4	*p*-Value
Villi Height (μm)	16.45 ^b^ ± 2.25	33.66 ^d^ ± 4.21	25.81 ^c^ ± 6.98	27.54 ^c^ ± 4.19	8.94 ^a^ ± 1.93	<0.0001

Values are the mean (*n* = 3) ± standard deviation. Different letters in the same row indicate significant statistical differences (*p* < 0.05). Newman–Keuls test.

**Table 5:** R and B cells from hepatopancreas histological samples of shrimp fed with experimental diets.

Treatment	CN	SP05	SP1	SP2	SP4	*p*-Value
R Cells	5.84 ^b^ ± 1.41	4.71 ^a^ ± 0.86	4.45 ^a^ ± 0.85	4.32 ^a^ ± 0.6	4.84 ^a^ ± 0.69	<0.0001
B Cells	1.7 ^b^ ± 2.03	0.32 ^a^ ± 0.70	0.13 ^a^ ± 0.34	0.1 ^a^ ± 0.31	0.19 ^a^ ± 0.40	<0.0001

Values are the mean (*n* = 3) ± standard deviation. Different letters in the same row indicate significant statistical differences (*p* < 0.05). Newman–Keuls test.

## Data Availability

The original contributions presented in this study are included in the article. Further inquiries can be directed to the corresponding author.
